# End-to-end decentralized formation control using a graph neural network-based learning method

**DOI:** 10.3389/frobt.2023.1285412

**Published:** 2023-11-07

**Authors:** Chao Jiang, Xinchi Huang, Yi Guo

**Affiliations:** ^1^ Department of Electrical Engineering and Computer Science, University of Wyoming, Laramie, WY, United States; ^2^ Department of Electrical and Computer Engineering, Stevens Institute of Technology, Hoboken, NJ, United States

**Keywords:** distributed multi-robot control, multi-robot learning, graph neural network, formation control and coordination, autonomous robots

## Abstract

Multi-robot cooperative control has been extensively studied using model-based distributed control methods. However, such control methods rely on sensing and perception modules in a sequential pipeline design, and the separation of perception and controls may cause processing latencies and compounding errors that affect control performance. End-to-end learning overcomes this limitation by implementing direct learning from onboard sensing data, with control commands output to the robots. Challenges exist in end-to-end learning for multi-robot cooperative control, and previous results are not scalable. We propose in this article a novel decentralized cooperative control method for multi-robot formations using deep neural networks, in which inter-robot communication is modeled by a graph neural network (GNN). Our method takes LiDAR sensor data as input, and the control policy is learned from demonstrations that are provided by an expert controller for decentralized formation control. Although it is trained with a fixed number of robots, the learned control policy is scalable. Evaluation in a robot simulator demonstrates the triangular formation behavior of multi-robot teams of different sizes under the learned control policy.

## 1 Introduction

The last decade has witnessed substantial technological advances in multi-robot systems, which have enabled a vast range of applications including autonomous transportation systems, multi-robot exploration, rescue, and security patrols. Multi-robot systems have demonstrated notable advantages over single-robot systems, such as enhanced efficiency in task execution, reconfigurability, and fault tolerance. In particular, the capacity of multi-robot systems to self-organize via local interaction gives rise to various multi-robot collective behaviors (e.g., flocking, formation, area coverage) that can be implemented to achieve team-level objectives (Guo, 2017).

A plethora of control methods have contributed to the development of multi-robot autonomy, which enables complex collective behaviors in multi-robot systems. One major control design paradigm focuses on decentralized feedback control methods (Panagou et al., 2015; Cortés and Egerstedt, 2017; Bechlioulis et al., 2019), which provide provable control and coordination protocols that can be executed efficiently at runtime. Control protocols are designed to compute robot actions analytically using the robot’s kinematic/dynamic model and communication graph, which specifies the interaction connectivity for local information exchange. Such hand-engineered control and coordination protocols separate the problem into a set of sequentially executed stages, including perception, state measurement/estimation, and control. However, this pipeline of stages may suffer from perception and state estimation errors that compound through the individual stages in the sequence (Zhang and Scaramuzza, 2018; Loquercio et al., 2021). Moreover, such a pipeline introduces latency between perception and actuation, as the time needed to process perceptual data and compute control commands accumulates (Falanga et al., 2019). Both compounding errors and latency are well-known issues that impact task performance and success in robotics. Learning-based methods, as another control design paradigm, have proven to be successful in learning control policies from data (Kahn et al., 2018; Devo et al., 2020; Li et al., 2020; Li et al., 2022). In particular, owing to the feature representation capability of deep neural networks (DNNs), control policies can be modeled to synthesize control commands directly from a raw sensor observations. Such control policies are trained to model an end-to-end computation that encompasses the traditional pipeline of stages and their underlying interactions (Loquercio et al., 2021).

Multi-robot learning has long been an active research area (Stone and Veloso, 2000; Gronauer and Diepold, 2021). Nonetheless, only in recent years, with the advancement of deep reinforcement learning techniques, has it become possible to handle challenges originating from real-world complexities. Breakthroughs have been made in computational methods that address long-standing challenges in multi-robot learning, such as non-stationarity (Foerster et al., 2017; Lowe et al., 2017; Foerster et al., 2018), learning to communicate (Foerster et al., 2016; Sukhbaatar and Fergus, 2016; Jiang and Lu, 2018), and scalability (Gupta et al., 2017). Various multi-robot control problems, such as path planning (Wang et al., 2021; Blumenkamp et al., 2022) and coordinated control (Zhou et al., 2019; Agarwal et al., 2020; Tolstaya et al., 2020a; Tolstaya et al., 2020b; Jiang and Guo, 2020; Kabore and Güler, 2021; Yan et al., 2022), have been tackled using learning-based methods. Despite the remarkable progress that has been made in multi-robot learning, the best approaches to architecture design and learning for scalable computational models that accommodate emerging information structures remain an open question. For example, it has yet to be understood what information should be dynamically gathered, and how this should be achieved, given a distributed information structure that only allows local inter-robot interaction. Recently, graph neural networks (GNNs) (Scarselli et al., 2008) have been used to model the structure for information-sharing between robots. A GNN can be trained to capture task-relevant information to be propagated and shared within the robot team via local inter-robot communication. GNNs have become an appealing framework for modeling of distributed robot networks (Agarwal et al., 2020; Zhou et al., 2019; Tolstaya et al., 2020a; b; Wang and Gombolay, 2020; Blumenkamp et al., 2022) due to their scalability and permutation-invariance (Gama et al., 2020).

In this article, we study a multi-robot formation problem using a learning-based method to find decentralized control policies that operate on robot sensor observations. The formation problem is defined for the multi-robot team to achieve triangular formations that constitute a planar graph with prescribed equidistant edge lengths. We use a GNN to model inter-robot communication for learning of scalable control policies. The GNN is combined with a convolutional neural network (CNN) to process sensor-level robot observations. Utilizing a model-based decentralized controller for a triangular formation as an expert control system, we train the deep neural network (DNN) with a data aggregation training scheme. We demonstrate in a robot simulator that the learned decentralized control policy is scalable to different sizes of multi-robot teams while being trained with a fixed number of robots.

The main contribution of this article is the demonstration of GNN-based end-to-end decentralized control for a multi-robot triangular formation. Compared to our prior work (Jiang et al., 2019) on learning-based end-to-end control of multi-robot formations, the approach presented in this article achieves a decentralized scalable control policy, while our prior work (Jiang et al., 2019) adopts centralized training mechanisms; additionally, the trained policy is not scalable and applies to a three-robot formation only. Compared to the recent GNN-based flocking control method (Tolstaya et al., 2020a), the triangular formation studied in this paper imposes additional geometric constraints for multi-robot coordinated motion as opposed to flocking behavior. Furthermore, our decentralized control scheme is end-to-end and takes robot LiDAR sensor data directly as input, while the method described by Tolstaya et al. (2020a) takes state values of robot positions as input. As mentioned earlier, end-to-end learning facilitates direct learning from sensor data, and can avoid the potential for compounding errors and latency issues commonly found in conventional designs in which perception and control are separated into sequential stages of a pipeline.

The remainder of this article is organized as follows. Section 2 presents the model of differential-drive mobile robots and the formulation of our multi-robot cooperative control problem. The GNN-based training and online control methods are described in Section 3. Robot simulation results are presented in Section 4. Section 5 discusses the main differences in comparison to existing learning-based methods. Finally, the article is concluded in Section 6.

## 2 Problem statement

In this paper, we consider a multi-robot cooperative control problem with *N* differential-drive mobile robots. The kinematic model of each robot *i* ∈ {1, …, *N*} is given by the discrete-time model:

$$\left[\begin{array}{c} x_{i}\left(t+1\right)\\ y_{i}\left(t+1\right)\\ \theta_{i}\left(t+1\right) \end{array}\right]=\left[\begin{array}{c} x_{i}\left(t\right)\\ y_{i}\left(t\right)\\ \theta_{i}\left(t\right) \end{array}\right]+G\left(t\right)\cdot \left[\begin{array}{c} u_{iL}\left(t\right)\\ u_{iR}\left(t\right) \end{array}\right],$$ (1)

where ${\left[x_{i},y_{i},\theta_{i}\right]}^{T}\in R^{3}$ is a robot state vector consisting of the position $p_{i}≜{\left[x_{i},y_{i}\right]}^{T}$ and the orientation *θ*_*i*_, and $u_{i}≜{\left[u_{iL},u_{iR}\right]}^{T}\in R^{2}$ is a control vector, with *u*_*iL*_ and *u*_*iR*_ representing left and right motor control, respectively. The matrix ***G*** (*t*) is defined as:

$$G\left(t\right)=\left[\begin{array}{cc} \frac{\Delta T}{2}\cos\;\theta_{i}\left(t\right) & \frac{\Delta T}{2}\cos\;\theta_{i}\left(t\right)\\ \frac{\Delta T}{2}\sin\;\theta_{i}\left(t\right) & \frac{\Delta T}{2}\sin\;\theta_{i}\left(t\right)\\ -\frac{\Delta T}{l} & \frac{\Delta T}{l} \end{array}\right],$$ (2)

where Δ*T* is the sampling period and *l* is the distance between the robot’s left and right wheels.

**FIGURE 1:**
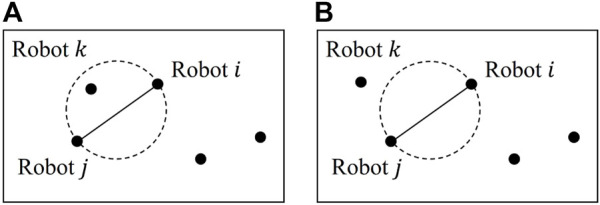
Gabriel graph: **(A)** robots *i* and *j* are not neighbors, as robot *k* exists in the circle whose diameter is defined by the distance between robots *i* and *j*; **(B)** robots *i* and *j* are valid neighbors, as there are no other robots in the circle.

We assume that each robot is equipped with a LiDAR sensor to detect neighboring robots. LiDAR measurements are transformed to an occupancy map, denoted by ***o***_*i*_(*t*), serving as a representation of the robot’s local observations. The proximity graph of the robot team is defined as a Gabriel graph (Mesbahi and Egerstedt, 2010), denoted as G=(V,E) , where *V* = {*v*_1_, …, *v*_*N*_} is the set of vertices corresponding to the robots located at $p_{1},\ldots ,p_{N}\in R^{2}$ and *E* is the set of edges. The line connecting the vertices *v*_*i*_, *v*_*j*_ ∈ *V*, *i* ≠ *j*, is said to be an edge if and only if the circle of diameter ‖ ***p***_*i*_ − ***p***_*j*_‖ containing both vertices *v*_*i*_ and *v*_*j*_ does not contain any vertex in its interior. An example of a valid and an invalid edge of a Gabriel graph can be seen in Figure 1. Robots *i* and *j*, associated with vertices *v*_*i*_ and *v*_*j*_, respectively, are said to be neighbors and can communicate if {*v*_*i*_, *v*_*j*_} ∈ *E*. Note that the proximity graph G is time-varying, as a robot’s neighbors vary when they move around.

The objective of cooperative control is to find a decentralized control protocol for each robot such that, starting from any initial positions in which there is at least one robot within the neighborhood of each other robot (i.e., the initial proximity graph G is connected), the group of robots achieves triangular formations with a prescribed inter-robot distance, d*, for all pairs of robots {*v*_*i*_, *v*_*j*_} ∈ *E*. That is, ‖ ***p***_*i*_ − ***p***_*j*_‖ → d* as *t* → *∞*, *∀*(*v*_*i*_, *v*_*j*_) ∈ *E*.

To address the multi-robot coordination problem as formulated in this way, we propose a learning-based method to find a decentralized and scalable control policy that can be deployed for each robot. A GNN in conjunction with a CNN will be used as the parameterized representation of the control policy. The neural network policy is decentralized in the sense that only local information obtained by each robot is used to compute a control action. We show in simulation experiments that, owing to the scalability of the GNN representation, the learned control policy is scalable in that, once trained with a given number of robots, the policy is applicable to different sizes of robot team if the team size remains unchanged during operation. In the next section, we introduce the architecture and training of the neural network control policy.

## 3 Methods

### 3.1 Overview of learning-based cooperative control

**FIGURE 2:**
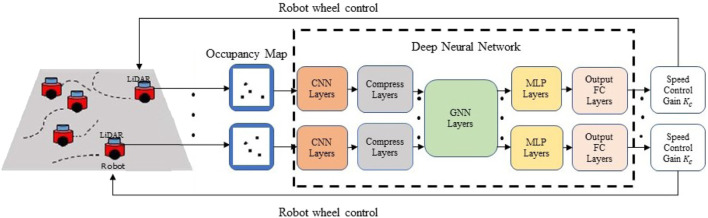
An overall diagram of end-to-end GNN-based decentralized formation control.

An overview of the proposed system for learning-based multi-robot cooperative control is shown in Figure 2. The decentralized control policy is parameterized by a DNN consisting of a CNN, a GNN, a multi-layer perceptron (MLP) network, and a fully connected (FC) network, as shown in the dashed box. The CNN extracts task-relevant features from an occupancy map obtained by the robot’s own onboard LiDAR sensor. The features from the robot’s local observations are communicated via the GNN, which models the underlying communication for information propagation and aggregation in the robot network. Given the features aggregated locally via the GNN, the MLP and FC layers compute a robot control command as the final output. The DNN policy can be expressed by

$$u_{i}=\pi \left(o_{i};G,\Theta \right),\;\forall i\in \left\{1,\ldots ,N\right\}.$$ (3)

To compute a control action ***u***_*i*_, the policy (3) uses each robot’s own observation ***o***_*i*_ and the local information aggregated from current neighboring robots, determined by the proximity graph G , that the GNN has access to. **Θ** is the tensor of parameters of the DNN, which is tuned during policy training. During online control, the DNN policy computes robot control end-to-end through a feed-forward pass in a decentralized manner. It is worth noting that the GNN block shown in Figure 2 represents data exchange within the entire robot team through local communication and does not signify a central communication unit. The computation of the GNN is decentralized, as each robot aggregates local information from its neighbors only. More details of the computation of the GNN are presented in Section 3.2.

We train the policy (3) via learning from demonstrations (LfD), and a model-based controller is used as an expert controller to provide demonstration data. The dataset is composed of pairs of robot observation ***o***_*i*_ and expert control action $u_{i}^{*}$ associated with that observation. Policy training then amounts to identification of the optimal parameters **Θ** that minimize the following loss function (for a single data sample):

$$L\left(\Theta \right)=\frac{1}{2}\|\pi \left(o_{i};G,\Theta \right)-u_{i}^{*}{\|}^{2}.$$ (4)

The loss function measures the difference between the neural network controller’s output, given by $\pi \left(o_{i};G,\Theta \right)$ , and the expert controller’s output $u_{i}^{*}$ computed under the same system state under which the observation ***o***_*i*_ is obtained. Minimizing the loss function encourages the policy to imitate the control strategies of the model-based decentralized controller.

### 3.2 Graph neural network

The feature vector $x_{i}\in R^{F}$ extracted by the CNN for each robot *i* will be communicated to the neighbors of that robot by one-hop communication via the GNN. The GNN architecture adopted in this article is the aggregation GNN (Gama et al., 2018). Each layer of the GNN performs a graph convolution that aggregates information from neighboring robots. Information aggregation with *k*-hop communication within the robot network at time step *t* creates a signal:

$$Z\left(t\right)=\left[X\left(t\right),SX\left(t\right),S^{2}X\left(t\right),\ldots ,S^{k}X\left(t\right)\right]\in R^{N\times F\left(k+1\right)},$$

where $X\left(t\right)={\left[x_{1}\left(t\right),x_{2}\left(t\right),\ldots ,x_{N}\left(t\right)\right]}^{T}\in R^{N\times F}$ is the collection of feature vectors of all robots and $S\left(t\right)\in R^{N\times N}$ is the graph shift operator (GSO) (Gama et al., 2018). The GSO is defined as a local linear operation applied to graph signals (e.g., the feature vector **x**_*i*_(*t*)) (Gama et al., 2018). Specifically, the *f*-th element of the feature vector for robot *i* after application of the GSO with one-hop communication (i.e., *k* = 1) is given by:

$${\left[SX\left(t\right)\right]}_{if}=\overset{N}{\underset{j=1}{\sum }}{\left[S\right]}_{ij}{\left[X\left(t\right)\right]}_{jf}.$$ (5)

The GSO is associated with the graph structure, and in our problem it is defined as the adjacency matrix, i.e., ${\left[S\right]}_{ij}=1$ if robot *i* and *j* are neighbors, otherwise ${\left[S\right]}_{ij}=0$ .

Each row *i* of **Z**(*t*), denoted by $z_{i}\in R^{F\left(k+1\right)}$ , is a local signal representing the information vector aggregated on the *i*th robot. The local signal **z**_*i*_ is then convolved with a bank of *FG* filters, denoted by $h\in R^{F\left(k+1\right)\times G}$ , to produce an output feature vector $y_{i}=\sigma_{\text{gnn}}\left(h^{T}z_{i}\right)\in R^{G}$ , where ***σ***_gnn_ (⋅) is a point-wise non-linear activation function. The elements of **h** represent the learnable filter weights, which are shared by all robots. The local feature vector, **y**_*i*_, is fed into the MLP and FC layers of each robot’s local policy to compute a control command for that robot. More details of the aggregation GNN used in this paper can be found in Gama et al. (2018). Note that we specifically use one-hop communication (i.e., *k* = 1) and set the number of GNN layers to one (i.e., the graph convolution operation ***σ***_gnn_ (⋅) is only performed once per time step) to reduce the communication load at each time step.

It is worth mentioning that our proposed policy model inherits the scalability property of the GNN. The scalability of GNNs stems from their permutation equivariance property and their stability to changes in topology (Gama et al., 2020). These properties allow GNNs to generalize signal processing protocols learned at local nodes to every other node with a similar topological neighborhood.

### 3.3 Policy training

#### 3.3.1 Expert controller

A model-based controller (Mesbahi and Egerstedt, 2010) for the multi-robot triangular formation problem was employed as the expert controller to provide training data. The expert controller achieves triangular formations by minimizing the potential function associated with robots *i* and *j*, i.e.,

$$U_{ij}=\frac{1}{2}{\left(\|p_{i}-p_{j}\|-d^{*}\right)}^{2},\;\forall \left\{i,j\right\}\in E.$$ (6)

The potential function takes its minimum value at the prescribed inter-robot distance, d*. Assuming single-integrator dynamics of the robots, i.e., ${\dot{p}}_{i}=v_{i}$ , the control law is given by

$$v_{i}=-K_{c}\underset{j\in N_{i}}{\sum }\frac{\|p_{i}-p_{j}\|-d^{*}}{\left\|p_{i}-p_{j}\right\|}\cdot \left(p_{i}-p_{j}\right),$$ (7)

where robot *j* belongs to the neighbors of robot *i*, $N_{i}$ (defined by the Gabriel graph), and *K*_*c*_ is the control gain. When the inter-robot distance is greater than d*, the controller exerts attractive force through the positive weight $\frac{\|p_{i}-p_{j}\|-d^{*}}{\left\|p_{i}-p_{j}\right\|}$ . When the inter-robot distance is smaller than d*, the controller repels the robots from one another as the weight becomes negative. At convergence, neighboring robots form triangular formations with distance d*.

The control input **v**_*i*_, computed by the expert controller for the single-integrator model, is converted to the motor control of the differential-drive robot model, ***u***_*i*_, using a coordinate transformation method (Chen et al., 2019). The transformation is given by

$$\left[\begin{array}{c} u_{iL}\\ u_{iR} \end{array}\right]=\left[\begin{array}{cc} \sin\;\theta_{i}+\frac{l}{2c}\cos\;\theta_{i} & \sin\;\theta_{i}-\frac{l}{2c}\cos\;\theta_{i}\\ -\sin\;\theta_{i}+\frac{l}{2c}\cos\;\theta_{i} & \sin\;\theta_{i}+\frac{l}{2c}\cos\;\theta_{i} \end{array}\right]\cdot v_{i},$$ (8)

where *l* is defined in (2) and *c* = *l*/2. The differential-drive robot (1) can then be controlled by the expert controller after transformation.

### 3.3.2 Policy training with DAgger

The DNN policy was trained via learning from demonstration, and a model-based controller was used to provide expert demonstration data. In order to obtain a model sufficiently generalizable to unseen states at test time, we used a Data Aggregation (DAgger) training framework (Ross et al., 2011). The idea behind this is that an empty dataset is gradually “aggregated” based on data samples with states visited by a learning policy and actions given by the expert. To this end, we selected the learning policy with a probability (1 − *β*) to execute a control action at each time step of data sample collection during training. The probability *β* was initialized to 1 and decayed by a factor of 0.9 after every 50 episodes.

The process of policy training with DAgger is outlined in Algorithm 1. As training progressed, the dataset D was aggregated with data samples in the form $\left(o_{i}\left(t\right),S\left(t\right),u_{i}^{*}\left(t\right)\right)$ . Since computation of a control input by the model at time *t* requires the robot’s local observation ***o***_*i*_(*t*) and the information aggregated through the GSO, we recorded ***S*** (*t*) along with the observation–action pair ***o***_*i*_(*t*) and $u_{i}^{*}\left(t\right)$ to create each data sample. In each training episode, mini-batches of size *B* were sampled from the dataset D to train our model by backpropagation of the mini-batch gradient of the loss calculated using (4).

Algorithm 1 Policy training with DAgger.

**Require:** Observation ***o***_*i*_(*t*), graph shift operator ***S*** (*t*), expert control action $u_{i}^{*}\left(t\right)$ at each time step *t*
**Ensure:** DNN policy $\pi \left(o_{i};G,\Theta \right)$

 1:  Initialize dataset D←∅

 2:  Initialize policy parameters **Θ** ←**Θ**_0_
 3:  Initialize *β* ← 1
 4:  **for** episode *e* = 1 to *E* **do**
 5:   Initialize robot state [*x*_*i*_, *y*_*i*_, *θ*_*i*_], *∀i* ∈ {1, …, *N*}
 6:   **for** time step *t* = 1 to *T* **do**
 7:    **for** robot *i* = 1 to *N* **do**
 8:     Query an expert control $u_{i}^{*}\left(t\right)\leftarrow \pi^{*}\left(s_{i}\left(t\right)\right)$

 9:     Get sample $\left(o_{i}\left(t\right),S\left(t\right),u_{i}^{*}\left(t\right)\right)$

 10:     Choose a policy ***π***_*i*_ ← *β* ***π*** * + (1 − *β*) ***π***
 11:    **end** **for**
 12: $D\leftarrow D\cup {\left\{\left(o_{i}\left(t\right),S\left(t\right),u_{i}^{*}\left(t\right)\right)\right\}}_{i=1}^{N}$

 13:    Execute policy ***π***_*i*_, *∀i* ∈ {1, …, *N*} to advance the environment
 14:   **end for**
 15:   **for** *n* = 1 to *K* **do**
 16:    Draw mini-batch samples of size *B* from D

 17:    Update policy parameters **Θ** by mini-batch gradient descent with loss (4)
 18:   **end for**
 19:   Update *β* ← 0.9*β* if mod (*e*, 50) = 0
 20:  **end for**
 21:  **return** learned policy $\pi \left(o_{i};G,\Theta \right)$

### 3.4 Online cooperative control

At test time (i.e., online formation control), a local copy of the learned policy $\pi \left(o_{i};G,\Theta \right)$ was deployed to each robot as a decentralized controller. At each time step, the local policy received an occupancy map and a control action was calculated in a feed-forward pass. One-hop communication was performed between neighboring robots *i* and *j*, for which {*v*_*i*_, *v*_*j*_} ∈ *E*, to aggregate information in a decentralized manner. Note that the communication graph is defined in Section 2 as a Gabriel graph and shown in Figure 1. The online cooperative control is outlined in Algorithm 2.

Algorithm 2 Online cooperative control.

**Require:** Occupancy map ***o***_*i*_(*t*)
**Ensure:** Robot control action ***u***_*i*_(*t*)
 1:  Initialize robot state [*x*_*i*_, *y*_*i*_, *θ*_*i*_], *∀i* ∈ {1, …, *N*}
 2:  **for** time step *t* = 1 to *T* **do**
 3:   **for** robot *i* = 1 to *N* **do**
 4:    Obtain an occupancy map ***o***_*i*_(*t*)
 5:    Aggregate information locally by applying (5) via one-hop communication
 6:    Compute a control action $u_{i}\left(t\right)\leftarrow \pi \left(o_{i};G,\Theta \right)$

 7:   **end for**
 8:   Execute the control action ***u***_*i*_, *∀i* ∈ {1, …, *N*} to advance the environment
 9:  **end for**

## 4 Experimental results

### 4.1 Simulation environment

The robot control simulation was conducted using the robot simulator CoppeliaSim (from the creators of V-REP). We chose a team of P3-DX mobile robots, each of which had a Velodyne VLP 16 LiDAR sensor used to obtain LiDAR data, which were then converted to occupancy maps. The LiDAR sensors were set to a sensing range of 10 m. The size of the occupancy map created from the sensor readings was 100 pixels × 100 pixels, making the granularity of the occupancy maps 0.1 m/pixel. The robot simulator was controlled via various Python scripts, as the simulator API can be accessed via local data communication to and from the client Python program.

The results were simulated using a computer with an Intel i7 12900K, 12-core CPU that ran at 3.6 GHz. The GPU used for rendering and neural network training and testing was an NVIDIA Titan Xp GPU. The PyTorch framework handled the GNN implementation as well as computations for training and testing of the neural network.

### 4.2 DNN implementation

The implementation details of the neural network architecture shown in Figure 2 are given in Table 1. The CNN layers were composed of multiple convolutional blocks. The input size of the first convolutional block was set to (1,100,100) to fit the size of the occupancy map. The features extracted from the input by the CNN were flattened into a vector of size (1,18432), which was further compressed by the compression block into a feature vector of size (1,128). That is, the dimension *F* of **x**_*i*_ was set to 128. The compressed feature vector was fed to the GNN block and communicated to neighboring robots. The GNN consisted of 1 graph convolution layer that produced a new feature vector of the same size as the input. That is, the dimension *G* of **y**_*i*_ was also set to 128. Muliple MLP blocks took the feature vector as input and output the robot control action, with dimensions (1,2).

**TABLE 1:** Blocks and parameters of the DNN.

Layer block	Input size	Output size
Convolutional Max Pool Block 1	(1,100,100)	(32,50,50)
Convolutional Block 2	(32,50,50)	(32,50,50)
Convolutional Max Pool Block 3	(32,50,50)	(64,25,25)
Convolutional Block 4	(64,24,24)	(128,12,12)
Convolutional Max Pool Block 5	(128,12,12)	(128,12,12)
Feature Compression Block 1	(1,18432)	(1,128)
GNN Block 1	(1,128)	(1,128)
MLP Block 1	(1,128)	(1,128)
MLP Block 2	(1,128)	(1,128)
Output MLP Block 3	(1,128)	(1,2)

### 4.3 System parameters and performance metrics

The desired triangular formation was set to d* = 2 m. We set *k* = 1 for the *k*-hop communication. The initial conditions in terms of robot positions were randomly generated in a circle of radius 5m, and the initial orientation of each robot was randomly chosen from the range [0, 2*π*]. The distance *l* between the robot’s left and right wheels was 0.331 m.

We trained the DNN on a team of five robots. To evaluate the performance of the trained model, we tested it on different numbers of robots ranging from *N* = 4 to *N* = 9. We defined the formation error between neighboring robots *i* and *j* at time *t* as $E_{i,j}\left(t\right)=|\|p_{i}\left(t\right)-p_{j}\left(t\right)\|-d^{*}|$ . The group formation error at any time *t* was defined as $E_{g}\left(t\right)=\frac{1}{N}\sum_{j\in N_{i}}E_{i,j}\left(t\right)$ .

During training with *N* = 5, the data collection period of each training episode (i.e., lines 6–14 of Algorithm 1) ran for at most 200 s, and a data point was collected every 0.05 s. We terminated the simulation if the temporal average of $E_{g}\left(t\right)/d^{*}$ over the most recent 20 s was smaller than 5%. Note that when we selected the speed control gain *K*_*c*_ in the expert control system (7), there was a tradeoff between convergence speed and steady-state error. A large *K*_*c*_ means that the system converges to the triangular formation more quickly, but may cause the system to oscillate around the equilibrium. We chose an adaptive control gain *K*_*c*_ in (7): the value was set to 1 initially, and then after $E_{g}\left(t\right)/d^{*}<0.05$ , *K*_*c*_ was decreased to slow down the robots as they approached one another.

During testing, we considered the multi-robot system to be *converged* if the group formation error (i.e., the temporal average of the group formation error $E_{g}\left(t\right)/d^{*}$ over the most recent 20 s) was smaller than 5% or 10%. We defined three performance metrics, as follows.

1. *Success rate*: *Rate* = *n*_*success*_/*n* is the number of successful cases as a proportion of the total number of tested cases *n*. A simulation run is considered successful if it converges before the end of the simulation. We present success rates with 5% and 10% tolerance in Table 2.
2. *Convergence time*: *T*_*converge*_ is the time at which a simulation run converges. Specifically, this is the first time point at which the temporal average of the group formation error over the most recent 20 s reaches the 5% threshold and then continues to decrease.
3. *Group formation error* defined in percentage form: ${\bar{E}}_{g}/d^{*}$ , where ${\bar{E}}_{g}$ is defined as the temporal average of $E_{g}\left(t\right)$ over the last 20 s prior to the end of the simulation.

**TABLE 2:** Success rates over 100 runs.

Number of robots	4	5	6	7	8	9
Success rate % (5% tolerance)	100	100	98	90	78	64
Success rate % (10% tolerance)	100	100	100	100	100	100

### 4.4 Training

We trained the DNN with a five-robot team by running Algorithm 1. The data collection period of each training episode (i.e., lines 6–14) ran for at most 200 s, with a data point being collected every 0.05 s. The value of *β*, representing the probability of picking the neural network controller vs. the expert controller at every time step, started at *β* = 1 in episode *e* = 1, and was updated every episode with the formula for episode *e* as $\beta_{e}=0.9^{\left\lfloor \frac{e}{50}\right\rfloor }$ where ⌊⋅⌋ represents the floor operator. Thus, *β* decayed by a factor of 0.9 every 50 training episodes. The loss function used was the mean squared error between the stored expert control command and the control command that was returned by the learned model. During training, the RMSprop optimizer (Mustapha et al., 2020) was used. The learning rate was set to 0.0001 and the size of the mini-batch *B*, used to calculate the gradient, was set to 16. After 200 episodes of training, the weights were saved for testing.

### 4.5 Testing results

After training the neural network model with a five-robot team, we tested our system for end-to-end decentralized formation control for robot teams of varying sizes by running Algorithm 2. Random initial conditions were used for robot starting positions. We present the empirical and statistical results below.

**FIGURE 3:**
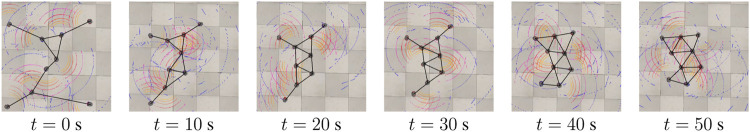
Snapshots showing online formation control of nine robots in the CoppeliaSim simulator. The colored arcs represent visualizations of LiDAR scanning.

**FIGURE 4:**
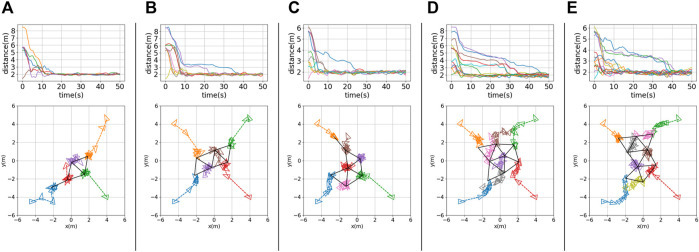
Testing results for **(A)** a five-robot team (*N* = 5), **(B)** a six-robot team (*N* = 6), **(C)** a seven-robot team (*N* = 7), **(D)** an eight-robot team (*N* = 8), and **(E)** a nine-robot team (*N* = 9). Top row: histories of inter-robot distance over time, $d_{ij}\left(t\right),\;i=1,\ldots ,N,j\in N_{i}$ . Bottom row: robot trajectories with robot positions (denoted by small colored triangles) sampled every 10 s; black solid lines indicate the final formation achieved.

Snapshots of nine robots achieving triangular formations in the CoppeliaSim simulator are shown in Figure 3. The solid black lines represent the formations achieved at different time steps. The testing results for different robot team sizes (*N* = 5, 6, 7, 8, 9) are shown in Figure 4, panels (a) to (e), respectively. The histories of inter-robot distance over time (i.e., $d_{ij}\left(t\right),\;i=1,\ldots ,N,j\in N_{i}$ ) and the trajectory of each robot are shown at the top and bottom of the figures, respectively. We can see that the robot team achieves the desired triangular formation and maintains the desired neighboring distance d* = 2 m. More simulation results for various robot team sizes can be found in the supplementary video file included with this article.

To evaluate *scalability*, we ran testing experiments with 100 different sets of initial conditions for each of the robot team sizes from 4 to 9. The success rates for different robot team sizes are shown in Table 2. We can see that the success rate reached 100% for any team size with 10% tolerance (i.e., the group formation error ${\bar{E}}_{g}/d^{*}$ is smaller than 10% as defined in Section 4.3). With the 5% tolerance threshold, the success rate decreased as the number of robots increased. This is due to the fact that when the robots approach the desired formation, small motion uncertainties cause oscillations in their trajectories, and these oscillations persist to a greater extent when the number of robots is higher. This phenomenon can be mitigated by reducing the speed control gain *K*_*c*_ further after the robots reach approximately the desired formation. However, tuning of this control parameter is tedious and must be achieved by trial and error. The success rates reported in this table were obtained using one set of *K*_*c*_. Thus, we can see that our method has good scalability: that is, although the model was trained with a five-robot team, the DNN policy can be applied to different sizes of robot team.

**FIGURE 5:**
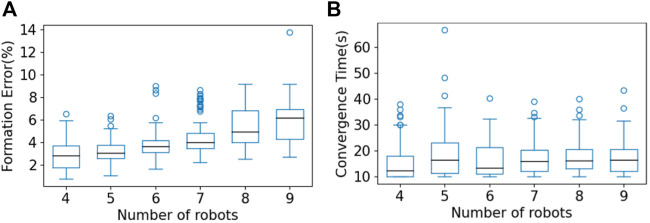
Box plots showing results for **(A)** group formation error and **(B)** convergence time for multi-robot teams of size 4 to 9, over 100 sets of initial conditions in multi-robot testing. The central mark in each box is the median; the edges of the boxes represent the 25th and 75th percentiles; the whiskers extend to the maximum/minimum; and the circles represent outliers.

To further evaluate performance, we tested 100 sets of initial conditions for each of the multi-robot teams of sizes 4 to 9. In Figure 5A, we present a box plot showing the group formation error ${\bar{E}}_{g}$ as defined in Section 4.3. We can see that the median formation errors are between 2% and 6% for robot team sizes ranging from 4 to 9. Figure 5B shows a box plot of the convergence time *T*_*converge*_. We can see that the median convergence time is around 15 s. Based on Figure 5, we can see that although the DNN model was trained with a five-robot team, the learned controller is scalable to other sizes of multi-robot team, and performance is satisfactory under the metrics of group formation error and convergence time.

**FIGURE 6:**
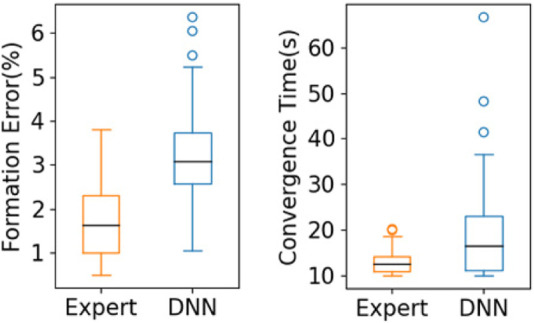
Comparison between the expert control system and our trained DNN model for the five-robot case under 100 sets of initial conditions.

To further compare the performance of the expert control system and that of our trained DNN models, we show a box plot representing results under 100 sets of initial conditions for the five-robot case in Figure 6. We can see that the expert control system achieved 1.76% for the median formation error and 12.9 s in median convergence time, while our DNN model achieved 3.12% and 18.2 s, respectively, indicating that the expert policy outperformed the end-to-end policy slightly. This is expected given that the expert policy, as defined in (7) and 8, uses the global position of the robots, which is assumed to be observable with perfect accuracy. The end-to-end policy, in contrast, uses LiDAR observations as input, which are noisy. It should be noted that the goal of the proposed method was not to outperform the expert controller given ideal state measurements. The main advantages of our method over the expert controller are that 1) the end-to-end computational model obtained by our method mitigates accumulation of error and latency in the traditional pipeline of computational modules used by the expert control method; and 2) our method does not need a localization system to obtain global robot positions, reducing overall system complexity.

### 4.6 Other formation shapes

The triangular formation control system that we designed can be extended to other formation shapes, such as line and circle formations. To examine such cases, we used additional landmarks (i.e., stationary robots positioned at pre-selected reference positions enabling other robots to achieve formation objectives) and modified the expert controller to achieve the desired formations.

**FIGURE 7:**
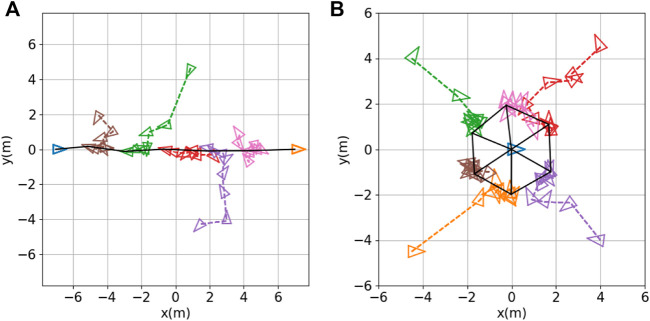
Other formation shapes: **(A)** line formation; **(B)** circle formation. Robot positions (denoted by small colored triangles) are sampled every 10 s. The black solid lines indicate the final formation achieved.

*Line formation:* The objective of the line formation is for the robots to position themselves in a line, at equal distances from one another, between two landmarks. We simulated a seven-robot team with two stationary robots serving as landmarks; these were positioned 14 m apart at each end of the desired line. The remaining five robots were controlled by the same expert controller (7) and (8) that we used for the triangular formation. We ran the same training algorithm (i.e., Algorithm 1) with the same hyperparameters as before. Figure 7A shows the results of testing, in which the robots achieved the desired line formation.

*Circle formation*: The objective of the circle formation is for *M* robots to position themselves in an *M*-sided regular polygonal formation with a landmark at the center. We simulated this using *M* = 6 robots, with one additional robot remaining stationary at the desired center position. We modified the expert controller (7) to the following:

$$\begin{array}{cc} v_{i}= & -K_{c}\underset{j\in N_{i}}{\sum }\frac{\|p_{i}-p_{j}\|-d^{*}}{\left\|p_{i}-p_{j}\right\|}\cdot \left(p_{i}-p_{j}\right)\\ & -K_{l}\frac{\|p_{i}-p_{l}\|-d^{*}}{\left\|p_{i}-p_{l}\right\|}\cdot \left(p_{i}-p_{l}\right), \end{array}$$ (9)

where d* = 2 is the radius of the circle, *p*_*l*_ = (0, 0) is the position of the center robot, and the control gains were set to *K*_*l*_ = 10, *K*_*c*_ = 1. We ran the same training algorithm (i.e., Algorithm 1) with the above modified expert control system, with the same hyperparameters as before. Figure 7B shows the results of testing, in which the six robots achieved the desired circle formation.

*Comparison with other work.* To empirically compare our method with other learning-based methods for formation control, we used the publicly available implementation of the work described by Agarwal et al. (2020) to evaluate performance for both line formation and circle formation over 100 trials. In the case of line formation, the mean group formation error ${\bar{E}}_{g}/d^{*}$ (as defined in Section 4.3) obtained by Agarwal et al. (2020) was 7.4% with standard deviation (SD) 2.9%; and using our method, the mean was 1.8% with SD 1.1%. In the case of circle formation, the formation error obtained by Agarwal et al. (2020) was 4.2% with SD 1.2%, and ours was 3.4% with SD 1.4%. Note that our simulation was implemented with realistic robot models in a robot simulator, while Agarwal et al. (2020) used a point mass robot model. Thus, our method achieved comparable or better formation error with more complicated robot models.

## 5 Discussion

To further compare our proposed method with recent work on learning-based multi-robot control, we summarize the main points of distinction in Table 3. Existing methods can be categorized as reinforcement learning (RL) (such as Agarwal et al. (2020); Li et al. (2022); Yan et al. (2022); Blumenkamp et al. (2022)) or learning from demonstration (LfD) (such as Li et al. (2020); Tolstaya et al. (2020a) and our work), depending on the training paradigm. The RL method does not require an expert policy, but its trial-and-error nature could make training intractable for multi-robot systems. The intractability issue is exacerbated when a realistic environment is considered, in which the dimensionalities of the robot state and observation spaces increase dramatically. As shown in the table, the RL-based methods (Blumenkamp et al., 2022; Li et al., 2022; Yan et al., 2022) were only validated for up to five robots when a realistic robot model was considered. It is noteworthy that Li et al. (2022) incorporated LfD into RL to mitigate the training intractability issue. Agarwal et al. (2020) used up to 10 robots for validation; however, the robot model was simplified as a point mass model. In contrast, our method employs an LfD paradigm that exploits expert demonstrations to guide the control policy search, thus considerably reducing the policy search space. Our method was validated for up to 9 robots with a realistic robot model and a high-dimensional observation space. Indeed, formation control of multi-robot systems has been well studied under the control regime using analytical model-based methods (Cortés and Egerstedt, 2017; Guo, 2017); the dynamic model-based expert controller used in this paper is mathematically provably correct and guarantees formation convergence of multi-robot systems (Mesbahi and Egerstedt, 2010).

**TABLE 3:** Summary of publications on learning-based multi-robot control.

Reference	Tasks	Method/architecture	Robot model	Policy input	# of robots trained (tested)	Scalability	Needs localization
Agarwal et al. (2020)	Coverage, line, Formation	RL/GNN	Point mass	Absolute pose	5 (2–10)	Yes	Yes
Li et al. (2022)	Path planning	RL + LfD/CNN + FC	Holonomic	LiDAR velocity, position	3–5 (3–5)	No	Yes
Yan et al. (2022)	Formation + path planning	RL/RNN	Ackermann steering	Distance angle	3–5 (3–5)	No	No
Blumenkamp et al. (2022)	Path planning	RL/GNN	Holonomic	Absolute pose	5 (5)	−	Yes
Li et al. (2020)	Path planning	LfD/CNN + GNN	Point mass	Binary map	4–12 (4–14)	Yes	No
Tolstaya et al. (2020a)	Flocking	LfD/GNN	Point mass	Absolute pose	100 (50–150)	Yes	Yes
**This paper**	Triangular formation	LfD/CNN + GNN	Nonholonomic	LiDAR	5 (3–9)	Yes	No

“−” represents a case in which no result was presented.

The scalability of a control policy can be evaluated by testing it with different numbers of robots than used in training. Among RL-based work, Yan et al. (2022) and Li et al. (2022) did not demonstrate the scalability of their methods. Agarwal et al. (2020) used a GNN architecture, but zero-shot generalizability (i.e., the results when a policy trained with a fixed number of robots is directly tested with a different number of robots) was low, as the success rate of the learned policy decreased when the number of robots in testing differed from that used in training. However, they showed that the scalability of this method can be improved when curriculum learning is exploited. In Li et al. (2020), Tolstaya et al. (2020a), and our work, GNN architectures with an LfD training paradigm are adopted; these have demonstrated a high level of scalability. Another advantage of our approach is that it does not need localization to obtain global information on the positions of the robots in order to compute control actions, as required in other work (Agarwal et al., 2020; Tolstaya et al., 2020a; Blumenkamp et al., 2022; Li et al., 2022).

## 6 Conclusion

In this article, we have presented a novel end-to-end decentralized multi-robot control system for triangular formation. Utilizing the capacity of a GNN to model inter-robot communication, we designed GNN-based algorithms for learning of scalable control policies. Experimental validation was performed in the robot simulator CoppeliaSim, demonstrating satisfactory performance for varying sizes of multi-robot teams. Future work will include implementation and testing on real robot platforms.

## Data Availability

The raw data supporting the conclusion of this article will be made available by the authors, without undue reservation.

## References

[B1] Agarwal A. Kumar S. Sycara K. Lewis M. (2020). “Learning transferable cooperative behavior in multi-agent teams,” in Proceedings of the 19th International Conference on Autonomous Agents and MultiAgent Systems, 1741–1743.

[B2] Bechlioulis C. P. Giagkas F. Karras G. C. Kyriakopoulos K. J. (2019). Robust formation control for multiple underwater vehicles. Front. Robotics AI 6, 90. 10.3389/frobt.2019.00090 PMC7805625 33501105

[B3] Blumenkamp J. Morad S. Gielis J. Li Q. Prorok A. (2022). “A framework for real-world multi-robot systems running decentralized gnn-based policies,” in IEEE International Conference on Robotics and Automation, 8772–8778.

[B4] Chen Z. Jiang C. Guo Y. (2019). “Distance-based formation control of a three-robot system,” in Chinese control and decision conference, 5501–5507.

[B5] Cortés J. Egerstedt M. (2017). Coordinated control of multi-robot systems: a survey. SICE J. Control, Meas. Syst. Integration 10, 495–503. 10.9746/jcmsi.10.495

[B6] Devo A. Mezzetti G. Costante G. Fravolini M. L. Valigi P. (2020). Towards generalization in target-driven visual navigation by using deep reinforcement learning. IEEE Trans. Robotics 36, 1546–1561. 10.1109/tro.2020.2994002

[B7] Falanga D. Kim S. Scaramuzza D. (2019). How fast is too fast? the role of perception latency in high-speed sense and avoid. IEEE Robotics Automation Lett. 4, 1884–1891. 10.1109/lra.2019.2898117

[B8] Foerster J. Farquhar G. Afouras T. Nardelli N. Whiteson S. (2018). “Counterfactual multi-agent policy gradients,” in Proceedings of the AAAI Conference on Artificial Intelligence. vol. 32. 10.1609/aaai.v32i1.11794

[B9] Foerster J. Nardelli N. Farquhar G. Afouras T. Torr P. H. Kohli P. (2017). “Stabilising experience replay for deep multi-agent reinforcement learning,” in International Conference on Machine Learning, 1146–1155.

[B10] Foerster J. N. Assael Y. M. De Freitas N. Whiteson S. (2016). “Learning to communicate with deep multi-agent reinforcement learning,” in Advances in neural information processing systems, 2137–2145.

[B11] Gama F. Isufi E. Leus G. Ribeiro A. (2020). Graphs, convolutions, and neural networks: from graph filters to graph neural networks. IEEE Signal Process. Mag. 37, 128–138. 10.1109/msp.2020.3016143 33758487

[B12] Gama F. Marques A. G. Leus G. Ribeiro A. (2018). Convolutional neural network architectures for signals supported on graphs. IEEE Trans. Signal Process. 67, 1034–1049. 10.1109/tsp.2018.2887403

[B13] Gronauer S. Diepold K. (2021). Multi-agent deep reinforcement learning: a survey. Artif. Intell. Rev. 1–49, 895–943. 10.1007/s10462-021-09996-w

[B14] Guo Y. (2017). Distributed cooperative control: emerging applications. Wiley.

[B15] Gupta J. K. Egorov M. Kochenderfer M. (2017). “Cooperative multi-agent control using deep reinforcement learning,” in International Conference on Autonomous Agents and Multiagent Systems (Springer), 66–83.

[B16] Jiang C. Chen Z. Guo Y. (2019). “Learning decentralized control policies for multi-robot formation,” in IEEE/ASME International Conference on Advanced Intelligent Mechatronics (AIM), Hong Kong, China, 8-12 July 2019, 758–765. 10.1109/AIM.2019.8868898

[B17] Jiang C. Guo Y. (2020). Multi-robot guided policy search for learning decentralized swarm control. IEEE Control Syst. Lett. 5, 743–748. 10.1109/lcsys.2020.3005441

[B18] Jiang J. Lu Z. (2018). “Learning attentional communication for multi-agent cooperation,” in Proceedings of the International Conference on Neural Information Processing Systems, 7265–7275.

[B19] Kabore K. M. Güler S. (2021). Distributed formation control of drones with onboard perception. IEEE/ASME Trans. Mechatronics 27, 3121–3131. 10.1109/tmech.2021.3110660

[B20] Kahn G. Villaflor A. Ding B. Abbeel P. Levine S. (2018). “Self-supervised deep reinforcement learning with generalized computation graphs for robot navigation,” in IEEE International Conference on Robotics and Automation (IEEE), 5129–5136.

[B21] Li M. Jie Y. Kong Y. Cheng H. (2022). “Decentralized global connectivity maintenance for multi-robot navigation: a reinforcement learning approach,” in IEEE International Conference on Robotics and Automation (ICRA), Philadelphia, PA, USA, 23-27 May 2022 (IEEE), 8801–8807. 10.1109/ICRA46639.2022.9812163

[B22] Li Q. Gama F. Ribeiro A. Prorok A. (2020). “Graph neural networks for decentralized multi-robot path planning,” in IEEE/RSJ International Conference on Intelligent Robots and Systems (IROS), Las Vegas, NV, USA, 24 Oct.-24 Jan. 2021 (IEEE), 11785–11792. 10.1109/IROS45743.2020.9341668

[B23] Loquercio A. Kaufmann E. Ranftl R. Müller M. Koltun V. Scaramuzza D. (2021). Learning high-speed flight in the wild. Sci. Robotics 6, eabg5810. 10.1126/scirobotics.abg5810 34613820

[B24] Lowe R. Wu Y. Tamar A. Harb J. Abbeel P. Mordatch I. (2017). “Multi-agent actor-critic for mixed cooperative-competitive environments,” in Proceedings of the International Conference on Neural Information Processing Systems, 6382–6393.

[B25] Mesbahi M. Egerstedt M. (2010). Graph theoretic methods in multiagent networks. Princeton University Press.

[B26] Mustapha A. Mohamed L. Ali K. (2020). “An overview of gradient descent algorithm optimization in machine learning: application in the ophthalmology field,” in Smart applications and data analysis. Editors Hamlich M. Bellatreche L. Mondal A. Ordonez C. (Cham: Springer International Publishing), 349–359.

[B27] Panagou D. Stipanović D. M. Voulgaris P. G. (2015). Dynamic coverage control in unicycle multi-robot networks under anisotropic sensing. Front. Robotics AI 2, 3. 10.3389/frobt.2015.00003

[B28] Ross S. Gordon G. Bagnell D. (2011). “A reduction of imitation learning and structured prediction to no-regret online learning,” in Proceedings of the Fourteenth International Conference on Artificial Intelligence and Statistics (JMLR Workshop and Conference Proceedings), 627–635.

[B29] Scarselli F. Gori M. Tsoi A. C. Hagenbuchner M. Monfardini G. (2008). The graph neural network model. IEEE Trans. Neural Netw. 20, 61–80. 10.1109/tnn.2008.2005605 19068426

[B30] Stone P. Veloso M. (2000). Multiagent systems: a survey from a machine learning perspective. Aut. Robots 8, 345–383. 10.1023/a:1008942012299

[B31] Sukhbaatar S. Fergus R. (2016). Learning multiagent communication with backpropagation. Adv. Neural Inf. Process. Syst. 29, 2244–2252.

[B32] Tolstaya E. Gama F. Paulos J. Pappas G. Kumar V. Ribeiro A. (2020a). “Learning decentralized controllers for robot swarms with graph neural networks,” in Conference on Robot Learning (ICASSP), Toronto, ON, Canada, 6-11 June 2021 (IEEE), 671–682. 10.1109/ICASSP39728.2021.9414219

[B33] Tolstaya E. Paulos J. Kumar V. Ribeiro A. (2020b). “Multi-robot coverage and exploration using spatial graph neural networks,” in IEEE/RSJ International Conference on Intelligent Robots and Systems (IROS), Prague, Czech Republic, 27 Sept.-1 Oct. 2021 (IEEE), 8944–8950. 10.1109/IROS51168.2021.9636675

[B34] Wang Y. Yue Y. Shan M. He L. Wang D. (2021). Formation reconstruction and trajectory replanning for multi-uav patrol. IEEE/ASME Trans. Mechatronics 26, 719–729. 10.1109/tmech.2021.3056099

[B35] Wang Z. Gombolay M. (2020). Learning scheduling policies for multi-robot coordination with graph attention networks. IEEE Robotics Automation Lett. 5, 4509–4516. 10.1109/lra.2020.3002198

[B36] Yan Y. Li X. Qiu X. Qiu J. Wang J. Wang Y. (2022). “Relative distributed formation and obstacle avoidance with multi-agent reinforcement learning,” in IEEE International Conference on Robotics and Automation (ICRA), Philadelphia, PA, USA, 23-27 May 2022 (IEEE), 1661–1667. 10.1109/ICRA46639.2022.9812263

[B37] Zhang Z. Scaramuzza D. (2018). “Perception-aware receding horizon navigation for MAVs,” in IEEE International Conference on Robotics and Automation. (ICRA), Brisbane, QLD, Australia, 21-25 May 2018 (IEEE), 2534–2541. 10.1109/ICRA.2018.8461133

[B38] Zhou S. Phielipp M. J. Sefair J. A. Walker S. I. Amor H. B. (2019). “Clone swarms: learning to predict and control multi-robot systems by imitation,” in IEEE/RSJ International Conference on Intelligent Robots and Systems. (IROS), Macau, China, 3-8 Nov. 2019 (IEEE), 4092–4099. 10.1109/IROS40897.2019.8967824

